# Supplemental datasets for the examination of the revival of large consulting practices at the big 4 and audit quality

**DOI:** 10.1016/j.dib.2020.106132

**Published:** 2020-08-06

**Authors:** Dain C. Donelson, Matthew Ege, Andrew J. Imdieke, Eldar Maksymov

**Affiliations:** a Henry B. Tippie College of Business, University of Iowa, IA 52242, USA; b Mays Business School, 4353 Texas A&M University, College Station, TX 77843, USA; c Mendoza College of Business, University of Notre Dame, Notre Dame, IN 46556, USA; d WP Carey School of Business, School of Accountancy, Arizona State University, PO BOX 873606, Tempe, AZ 85287-3606, USA

**Keywords:** Audit Quality, Audit Regulation, Consulting, PCAOB

## Abstract

These data analyses have been co-submitted to *Accounting, Organizations, and Society* with the research article “The Revival of Large Consulting Practices at the Big 4 and Audit Quality ” [2]. The purpose of these data analyses is to assist readers of the research article in obtaining further detailed analyses performed therein regarding the channels through which audit quality is affected by consulting firm acquisitions. These analyses include 1) the timing of the effects of consulting firm acquisitions on audit quality; 2) the size of the consulting firm acquisition's effect on audit quality; 3) whether acquisitions differentially affect restatement breadth; 4) whether results are due to the PCAOB targeting offices that acquire consulting practices; and 5) whether consulting firm acquisitions affect national audit firm audit quality. These analyses can inform future research on audit quality by providing insights that may be useful in developing research ideas and performing extensions of these analyses. Some data used in these analyses are available via a subscription to the Wharton Research Data Service while other data are publicly available via search of Google, and, with subscription, via search of Factiva, and the Capital IQ database.

**Specifications Table**

**Table:** 

**Subject**	Accounting
**Specific subject area**	The Revival of Large Consulting Practices at the Big 4 and Audit Quality
**Type of data**	Memo Table
**How data were acquired**	Data were obtained from Wharton Research Data Services databases including Audit Analytics, Compustat, and CRSP. Data were also obtained from the Capital IQ Database. Data were also obtained by searching Google and Factiva.
**Data format**	Secondary Analyzed
**Parameters for data collection**	Data analyses 1 through 5 were performed using data compiled from Audit Analytics, Compustat and CRSP housed with Wharton Research Data Services. We also use data from the related research article where we identified U.S. Big 4 acquisitions of consulting firms from 2005-2015 by searching Google and Factiva for each of the Big 4 along with the keywords “consulting” and “acquisition.” We also downloaded Big 4 mergers and acquisitions from the Capital IQ database.
**Description of data collection**	Our data analyses in Tables 1 through Table 4 result from data sample selection described in Section 3.1 and Table 1 of the related research article. The sample includes all clients of U.S. offices of the Big 4 from 2006 to 2016 for which control variables can be calculated. Data come from Audit Analytics, Compustat, and CRSP house on Wharton Research Data Services.
**Data source location**	Wharton Research Data Services (https://wrds-www.wharton.upenn.edu/)
**Data accessibility**	Access to Audit Analytics, Compustat, and CRSP is available through subscription to Wharton Research Data Services (https://wrds-www.wharton.upenn.edu/). In the Appendix below, variable definitions are defined along with the relevant database from which the variable has been created. For example, to access variables from Audit Analytics, please click the link above for WRDS, login, and under “Subscriptions” click on “Audit Analytics”. From there, you can query for the appropriate variables. Sample selection procedures are detailed in the co-submitted manuscript to Accounting, Organizations, and Society. We have provided a temporary login to WRDS that expires on August 16, 2020. This link can be used for peer review, but can't be shared and should be used by a reviewer/editor who already has a subscription to WRDS. This temporary login must be removed from this DIB submission document once peer review is complete. The temporary login is: Username: dib2020 Password: datainbrief2020 Access to the list of Big 4 Consulting Acquisitions is available in Appendix A of the related research article.
**Related research article**	Donelson, D., M. Ege, A. Imdieke, and E. Maksymov. The Revival of Large Consulting Practices at the Big 4 and Audit Quality. Accounting, Organizations, and Society (In Press).

**Value of the Data**

Researchers on audit quality could benefit from these data by informing, motivating, and supplementing future research of how audit firm acquisitions and changes in audit firm culture affect audit office audit quality.
These datasets can inform future research on audit quality by providing insights that may be useful in developing research ideas and performing analysis especially related to audit quality.
Auditors can use these datasets and analyses to inform their decision process regarding future acquisitions.

## 1 Data description

Our data analyses in Tables 1 through Table 4 result from the data obtained and sample selection procedures described in Section 3.1 and Table 1 of the corresponding research article. The sample includes all clients of U.S. offices of the Big 4 from 2006 to 2016 for which control variables can be calculated. Data are available from Audit Analytics, Compustat, and CRSP via a subscription to Wharton Research Data Services. Data related to consulting acquisitions by the Big 4 are available in the related research article or by public search of Google and Factiva. Data related to Big 4 mergers and acquisitions are available via subscription to the Capital IQ database.

**Table 1:** OLS regressions of audit quality measures by acquisition type and year after acquisition

Independent variables	OLS regressions, dependent variable is listed in row below				
	*RESTATEMENTS*	*REISSUANCE_* *RESTATEMENTS*	*NON_REISSUANCE_* *RESTATEMENTS*	*ACCRUAL_* *QUALITY*	*ABNORMAL_* *ACCRUALS*
	(1)	(2)	(3)	(4)	(5)
*ERP_POST_t1*	−0.012	−0.004	0.000	0.007	−0.001
	*0.023*	*0.022*	*0.034*	*0.007*	*0.007*
*ERP_POST_t2*	0.029	−0.012	0.044	−0.001	−0.001
	*0.060*	*0.019*	*0.062*	*0.011*	*0.008*
*ERP_POST_t3*	−0.011	−0.015*	0.010	0.001	−0.010**
	*0.020*	*0.009*	*0.021*	*0.003*	*0.005*
*ERP_POST_t3+*	−0.046*	−0.015**	−0.033	0.000	−0.006
	*0.024*	*0.007*	*0.024*	*0.005*	*0.007*
*NON_ERP_POST_t1*	0.043*	0.027**	0.019	0.001	−0.003
	*0.024*	*0.012*	*0.023*	*0.003*	*0.005*
*NON_ERP_POST_t2*	0.038	−0.009	0.044**	−0.002	−0.007
	*0.024*	*0.008*	*0.022*	*0.003*	*0.005*
*NON_ERP_POST_t3*	0.095***	0.025**	0.078***	−0.004	0.000
	*0.029*	*0.011*	*0.029*	*0.004*	*0.006*
*NON_ERP_POST_t3+*	0.069***	0.010	0.065***	0.001	0.008
	*0.023*	*0.009*	*0.024*	*0.004*	*0.004*
*LN_MVE*	−0.005	−0.002	−0.002	0.000	0.000
	*0.003*	*0.001*	*0.003*	*0.000*	*0.001*
*ROA*	0.012	0.012	−0.001	−0.005	−0.037***
	*0.025*	*0.016*	*0.023*	*0.006*	*0.011*
*NEGATIVE_ROA*	0.014	0.003	0.010	−0.001	0.003
	*0.010*	*0.007*	*0.009*	*0.002*	*0.003*
*LEVERAGE*	0.038***	0.006	0.031***	−0.004	−0.006
	*0.013*	*0.007*	*0.011*	*0.003*	*0.004*
*BTM*	0.017***	0.004*	0.013***	−0.002***	−0.003***
	*0.004*	*0.002*	*0.003*	*0.001*	*0.001*
*FIN_RAISED*	0.015	0.014	0.002	0.030***	0.048***
	*0.020*	*0.013*	*0.017*	*0.004*	*0.005*
*STD_DEV_CFO*	−0.118**	−0.029	−0.102*	0.188***	0.230***
	*0.059*	*0.036*	*0.055*	*0.016*	*0.022*
*STD_DEV_SALE*	−0.001	0.012	−0.013	0.027***	0.034***
	*0.018*	*0.014*	*0.017*	*0.004*	*0.006*
*LOSS_PROPORTION*	0.010	0.015*	0.000	0.012***	0.006
	*0.015*	*0.009*	*0.014*	*0.003*	*0.004*
*INTANGIBLES*	0.005**	0.000	0.004**	−0.001***	−0.002***
	*0.002*	*0.001*	*0.002*	*0.000*	*0.000*
*MERGER*	0.002	0.001	0.003	0.007***	0.007***
	*0.007*	*0.004*	*0.007*	*0.001*	*0.001*
*SALES_GROWTH*	0.005	0.005	−0.001	0.010***	0.021***
	*0.007*	*0.005*	*0.006*	*0.002*	*0.003*
*MW*	0.393***	0.338***	0.160***	−0.001	0.002
	*0.017*	*0.018*	*0.021*	*0.001*	*0.002*
*404B_OPINION*	0.014	−0.006	0.017*	−0.002	0.000
	*0.010*	*0.009*	*0.010*	*0.001*	*0.001*
*PRIOR_RESTATEMENT*	0.042***	0.014***	0.033***	0.000	0.006*
	*0.008*	*0.004*	*0.007*	*0.003*	*0.004*
*NON_BUSYSEASON*	0.003	0.007	−0.003	−0.006*	−0.005
	*0.008*	*0.004*	*0.007*	*0.003*	*0.005*
Year Fixed Effects?	Yes	Yes	Yes	Yes	Yes
Industry Fixed Effects?	Yes	Yes	Yes	Yes	Yes
Auditor Office Fixed Effects?	Yes	Yes	Yes	Yes	Yes
Total Obs.	18,787	16,854	17,987	17,061	18,279
*R*^2^	0.10	0.16	0.06	0.16	0.14

This table reports the results of the following ordinary least squares regression:

*AUDIT_QUALITY* = β_0_+ β_1_*ERP _POST_t1*+ β_2_*ERP _POST_t2*+ β_3_*ERP _POST_t3*+ β_4_*ERP _POST_t3*+

Β_5_*NON_ ERP _POST_t1* + β_6_*NON_ ERP _POST_t2* + β_7_*NON_ ERP _POST_t3* + β_8_*NON_ ERP _POST_t3+* +

β’*CONTROLS + Industry Fixed Effects + Year Fixed Effects + Auditor Office Fixed Effects +* ε

*AUDIT_QUALITY* represents one of the audit quality measures listed below in columns (1) thru (5).*ERP_POST_tx (NON_ERP_POST_tx)* is an indicator set to 1 for any audit-office year audit with a signature *x* years following the first consulting practice acquisition made by the audit firm where the consulting firm's headquarters is in the city of the audit office and where the acquisition is (not) related to enterprise resource planning or analytics consulting. Detailed variable definitions are provided in the Appendix. Standard errors are in italics and are two-way clustered by company and auditor office. *, **, and *** represent statistical significance at the 10%, 5%, and 1% levels, respectively, using a two-tailed test.

## 2 Experimental design, materials, and methods

In compiling our data analyses, we identify U.S. Big 4 acquisitions of consulting firms from 2005-2015 by searching Google and Factiva for each of the Big 4 along with the keywords “consulting” and “acquisition.”^1^ We also download Big 4 mergers and acquisitions from the Capital IQ database.^2^ These data are presented in Appendix A of the related research article. Definitions and sources of our variables of interest and control variables are included in the Appendix herein.

## 3 Data analyses

### 3.1 Data analysis #1: the timing of the effects of consulting firm acquisitions on audit quality

We argue that a potential channel through which audit quality is affected by consulting firm acquisitions is through changes in office culture and expertise. When an audit office has to absorb a consulting firm office, the culture of the audit firm office will likely be affected and, depending on the acquisition type, the expertise of the audit firm too. However, the immediacy of the effects of culture and expertise may vary differ. While research on culture reports that cultural shifts tend to take place in less than a year after the merger [1], expertise research documents that effective expertise transfers at the firm-level may take longer when cultures of the two firms diverge significantly because convergence of cultures allows for a more effective transfer of expertise between firms [3].

To empirically test when consulting acquisitions begin to affect audit quality, we re-perform the analyses presented in Table 4 of the related research article after creating time-series indicators for each of the acquisition types. Specifically, we include *ERP_POST_t_j_* and *NON_ERP_POST_t_j_* which are separate indicator variables for each of the first, second, and third years following the consulting firm acquisition. We also include *ERP_POST_t_3+_* and *NON_ERP_POST_t_3+_,* which are indicators for all post acquisition observations greater than three years after the acquisition. The intuition behind these measures is to capture time-series effects of cultural change related to consulting firm acquisitions on audit quality.

Results are presented in Table 1 below. We find that improvements in audit quality after ERP-related consulting acquisitions are concentrated in year *t+3* and years greater than three after the acquisition. Specifically, the coefficient on *ERP_POST_t_3_* is negative and significant for reissuance restatements in column (2) and for abnormal accruals in column (5) (*p*<0.10) while the coefficient on *ERP_POST_t_3+_* is negative and significant for both restatements and reissuance restatements (*p*<0.10 and *p*<0.05, respectively). These results suggest that the positive effects of expertise generated from ERP-related acquisitions are realized gradually.

Conversely, the negative effects of non-ERP-related acquisitions on audit quality are more immediate. Specifically, the coefficient on *NON_ERP_POST_t_1_* is positive and significant for restatements and reissuance restatements (p<0.10 and p<0.05, respectively). In addition, we find an increase in the likelihood of non-reissuance misstatements in the second year after non-ERP-related consulting acquisitions (*p*<0.05). Further, the negative effect on audit quality persists over time for restatements and non-reissuance restatements as the coefficients in column (1) on *NON_ERP_POST_t_3_* and *NON_ERP_POST_t_3+_* are both positive and significant (*p*<0.01). We also find a positive and significant coefficient on *NON_ERP_POST_t_3+_* related to abnormal accruals (*p*<0.10), further suggesting the negative effects of non-ERP-related acquisitions persist.

Collectively, when analyzing the immediacy of the effect of consulting acquisitions on audit quality, we find that the benefits of ERP-related acquisitions start to occur in later years, suggesting that the benefits of learning and expertise are more gradual. However, the detriments to audit quality related to non-ERP-related acquisitions start to occur in the first year after the consulting acquisition, suggesting that audit office culture effects related to commercialism and client advocacy have more immediate effects.

### 3.2 Data analysis #2: size of consulting firm acquisition

The size of the effects we have documented above and in the related research article could be dependent on the size of the acquired consulting firm. Compared to a smaller consulting firm, senior executives from a larger consulting firm would likely have a greater effect on the culture of and absorption of expertise by the local audit office because these executives oversee a greater number of engagements and consultants. In other words, they likely have greater status and have more influence when they initially join the local audit office compared to executives from a smaller consulting firm. Also, a larger firm should bring more consultants to the local audit office than a smaller firm.

To empirically test whether consulting firm size affects results, we re-perform the analyses in Table 4 of the related research article after splitting acquisition variables by size. Specifically, we include indicator variables for Large and Small acquisitions, set to one when the ratio of the number of employees of the acquired consulting firm over the total audit fees for the nearest audit office in year of the acquisition is at or above (below) the median for all acquisitions, and 0 otherwise.^3^ The intuition is to capture the size of the consulting firm relative to the corresponding audit office, which will absorb the leaders and consultants of the consulting firm.^4^

Results are presented in Table 2 below. We find that both large and small non-ERP acquisitions are positively correlated with restatements and non-reissuance restatements whereas we do not find a significant correlation between large or small ERP acquisitions and restatements and non-reissuance restatements (columns (1) and (3)). In column (2), only large ERP acquisitions (large non-ERP acquisitions) are negatively (positively) correlated with reissuance restatements.^5^ However, in performing F-tests for differences between coefficients we do not find evidence that the coefficients are statistically different between large and small ERP-related acquisitions or between large and small non-ERP-related acquisitions in columns (1), (2), and (3).^6^

**Table 2:** OLS regressions of audit quality measures by acquisition type and acquisition size.

Independent variables	OLS regressions, dependent variable is listed in row below				
	*RESTATEMENTS*	*REISSUANCE_* *RESTATEMENTS*	*NON_REISSUANCE_* *RESTATEMENTS*	*ACCRUAL_* *QUALITY*	*ABNORMAL_* *ACCRUALS*
	(1)	(2)	(3)	(4)	(5)
*LARGE_ERP_POST*	−0.016	−0.017**	0.000	0.006	−0.006
	*0.024*	*0.007*	*0.021*	*0.005*	*0.006*
*SMALL_ERP_POST*	−0.026	−0.006	−0.014	−0.004	−0.005
	*0.033*	*0.014*	*0.038*	*0.004*	*0.004*
*LARGE_NON_ERP_POST*	0.067***	0.012*	0.056**	0.003	0.007
	*0.023*	*0.008*	*0.022*	*0.004*	*0.004*
*SMALL_NON_ERP_POST*	0.054**	0.012	0.047**	−0.002	−0.004
	*0.022*	*0.008*	*0.022*	*0.002*	*0.004*
*LN_MVE*	−0.005	−0.002	−0.002	0.000	0.000
	*0.003*	*0.001*	*0.003*	*0.000*	*0.001*
*ROA*	0.011	0.012	−0.002	−0.005	−0.037***
	*0.025*	*0.016*	*0.023*	*0.006*	*0.011*
*NEGATIVE_ROA*	0.014	0.003	0.010	−0.001	0.003
	*0.010*	*0.007*	*0.009*	*0.002*	*0.003*
*LEVERAGE*	0.038***	0.006	0.031***	−0.004	−0.006*
	*0.013*	*0.007*	*0.011*	*0.003*	*0.004*
*BTM*	0.017***	0.004*	0.013***	−0.002***	−0.003***
	*0.004*	*0.002*	*0.003*	*0.001*	*0.001*
*FIN_RAISED*	0.016	0.014	0.003	0.030***	0.048***
	*0.020*	*0.013*	*0.017*	*0.004*	*0.005*
*STD_DEV_CFO*	−0.120**	−0.029	−0.104*	0.188***	0.229***
	*0.059*	*0.037*	*0.055*	*0.016*	*0.022*
*STD_DEV_SALE*	−0.001	0.013	−0.013	0.027***	0.034***
	*0.018*	*0.014*	*0.016*	*0.004*	*0.006*
*LOSS_PROPORTION*	0.010	0.015*	−0.001	0.012***	0.006
	*0.015*	*0.009*	*0.014*	*0.003*	*0.004*
*INTANGIBLES*	0.005**	0.000	0.004**	−0.001***	−0.002***
	*0.002*	*0.001*	*0.002*	*0.000*	*0.000*
*MERGER*	0.003	0.001	0.003	0.007***	0.007***
	*0.007*	*0.004*	*0.007*	*0.001*	*0.001*
*SALES_GROWTH*	0.005	0.005	−0.001	0.010***	0.022***
	*0.007*	*0.005*	*0.006*	*0.002*	*0.003*
*MW*	0.393***	0.338***	0.160***	−0.001	0.002
	*0.017*	*0.018*	*0.021*	*0.001*	*0.002*
*404B_OPINION*	0.014	−0.006	0.017*	−0.002	0.000
	*0.011*	*0.009*	*0.010*	*0.001*	*0.001*
*PRIOR_RESTATEMENT*	0.042***	0.014***	0.034***	0.000	0.007*
	*0.008*	*0.004*	*0.007*	*0.003*	*0.004*
*NON_BUSYSEASON*	0.003	0.007	−0.003	−0.006*	−0.005
	*0.008*	*0.004*	*0.007*	*0.003*	*0.005*
Year Fixed Effects?	Yes	Yes	Yes	Yes	Yes
Industry Fixed Effects?	Yes	Yes	Yes	Yes	Yes
Auditor Office Fixed Effects?	Yes	Yes	Yes	Yes	Yes
Total Obs.	18,787	16,854	17,987	17,061	18,279
*R*^2^	0.10	0.16	0.06	0.16	0.08

This table reports the results of the following ordinary least squares regression:

*AUDIT_QUALITY* = β_0_+ β_1_*ERP _POST +* β_2_*LARGE_NON_ ERP_POST*+ β_3_*SMALL_NON_ ERP _POST* + β’*CONTROLS +*

*Industry Fixed Effects + Year Fixed Effects + Auditor Office Fixed Effects +* ε

*AUDIT_QUALITY* represents one of the audit quality measures listed below in columns (1) thru (5). *LARGE_ERP_POST (SMALL_ERP_POST)* is an indicator set to 1 for any audit-office year audit with a signature following the first consulting practice acquisition made by the audit firm where the consulting firm's headquarters is in the city of the audit office and where the number of employees acquired scaled by total audit fees for the related audit office in the year of consulting acquisition is at or above (below) the median of all consulting acquisitions, and where the acquisition is related to enterprise resource planning or analytics consulting, and 0 otherwise. *LARGE_NON_ERP_POST (SMALL_NON_ERP_POST)* is an indicator set to 1 for any audit-office year audit with a signature following the first consulting practice acquisition made by the audit firm where the consulting firm's headquarters is in the city of the audit office and where the number of employees acquired scaled by total audit fees for the related audit office in the year of consulting acquisition is at or above (below) the median of all consulting acquisitions, and 0 otherwise and where the acquisition is not related to enterprise resource planning or analytics consulting. Detailed variable definitions are provided in the Appendix. Standard errors are in italics and are two-way clustered by company and auditor office. *, **, and *** represent statistical significance at the 10%, 5%, and 1% levels, respectively, using a two-tailed test.

### 3.3 Data analysis #3: do acquisitions affect audit quality differentially? Evidence based on restatement breadth

Insights from our interviews with partners documented in the related research article suggest that ERP-related consulting acquisitions may especially reduce instances of restatements that affect multiple processes or accounts. The interviewees expressed that ERP expertise directly relates to deep knowledge of processes and related controls. Thus, when this expertise is used on an audit, it facilitates a better understanding of key processes and testing of key controls, which then result in the execution of appropriate substantive tests and the achievement of desired audit risk. Therefore, the likelihood of a broad misstatement (one that touches multiple accounts or processes) decreases. We examine this possibility in Table 3.

**Table 3:** OLS regressions of restatement data intensiveness by acquisition type

**Panel A** OLS regressions, dependent variable is listed in row below						
Independent variables	*NUM_RESTATEMENT_REASONS*					
	*RESTATEMENTS*	*REISSUANCE_* *RESTATEMENTS*	*NON_REISSUANCE_* *RESTATEMENTS*			
	(1)	(2)	(3)			
*ERP_POST*	−0.085**	−0.018	−0.063			
	*0.035*	*0.020*	*0.044*			
*NON_ERP_POST*	0.111***	0.050**	0.074**			
	*0.038*	*0.020*	*0.031*			
Controls?	Yes	Yes	Yes			
Year Fixed Effects?	Yes	Yes	Yes			
Industry Fixed Effects?	Yes	Yes	Yes			
Auditor Office Fixed Effects?	Yes	Yes	Yes			
Total Obs.	18,787	16,854	17,987			
R^2^	0.10	0.14	0.05			

**Panel B** OLS regressions, dependent variable is listed in row below						
	*AUDIT_QUALITY*					
	*RESTATEMENTS*		*REISSUANCE_* *RESTATEMENTS*		*NON_REISSUANCE_* *RESTATEMENTS*	
	2 or fewer restatement reasons	Above 2 restatement reasons	2 or fewer restatement reasons	Above 2 restatement reasons	2 or fewer restatement reasons	Above 2 restatement reasons
Independent variables	(1)	(2)	(3)	(4)	(5)	(5)
*ERP_POST*	−0.010	−0.020***	−0.010	−0.005	0.002	−0.015*
	*0.018*	*0.006*	*0.008*	*0.004*	*0.018*	*0.008*
*NON_ERP_POST*	0.055***	0.012	0.009*	0.006*	0.047***	0.006
	*0.017*	*0.008*	*0.005*	*0.004*	*0.017*	*0.007*
Controls?	Yes	Yes	Yes	Yes	Yes	Yes
Year Fixed Effects?	Yes	Yes	Yes	Yes	Yes	Yes
Industry Fixed Effects?	Yes	Yes	Yes	Yes	Yes	Yes
Auditor Office Fixed Effects?	Yes	Yes	Yes	Yes	Yes	Yes
Total Obs.	18,040	16,912	16,581	16,438	17,550	16,602
*R*^2^	0.07	0.09	0.10	0.12	0.04	0.04

This table reports the results of the following ordinary least squares regressions:

Panel A: *NUM_RESTATEMENT_REASONS* = β_0_+ β_1_*ERP _POST +* β_2_*NON_ ERP _POST* + β’*CONTROLS +*

*Industry Fixed Effects + Year Fixed Effects + Auditor Office Fixed Effects +* ε

Panel B: *AUDIT_QUALITY* = β_0_+ β_1_*ERP _POST +* β_2_*NON_ ERP _POST* + β’*CONTROLS +*

*Industry Fixed Effects + Year Fixed Effects + Auditor Office Fixed Effects +* ε

*NUM_RESTATEMENT_REASONS* represents the number of restatement reason codes generated by Audit Analytics for each restatement filed.

*AUDIT_QUALITY* represents one of the audit quality measures listed below in columns (1) thru (3) in Panel A (columns (1) thru (6) in Panel B).

In Panel B, each *AUDIT_QUALITY* proxy is partitioned by the median number of restatement reasons found in our sample (less than or equal to two and greater than two.

*ERP_POST (NON_ERP_POST)* is an indicator set to 1 for office-year-audits with an audit opinion signature after the first consulting practice acquisition made by the audit firm where the acquired consulting practice's headquarters is in the same city as the audit office and where the consulting practice acquisition is (not) related to enterprise resource planning consulting or analytics consulting, and 0 otherwise. Detailed variable definitions are provided in the Appendix. Standard errors are in italics and are two-way clustered by company and auditor office. *, **, and *** represent statistical significance at the 10%, 5%, and 1% levels, respectively, using a two-tailed test.

As a proxy for restatement breadth, we consider the number of accounts that the restatement affects. We re-estimate equation (1) from the related research article, using *NUM_RESTATEMENT_REASONS*, defined as the number of restatement codes identified by Audit Analytics as the dependent variable for all observations in our sample. A larger number of restatement codes represents a broader restatement because the restatement affects a larger number of accounts and processes. Results are presented in Panel A of Table 3. The coefficient on *ERP_POST* when examining the number of restatement reasons for all restatements is negative and significant (p=0.02) while the coefficient on *NON_ERP_POST* is positive and significant (p<0.01). Further, the coefficients on *NON_ERP_POST* are positive and significant when breaking the restatement type between reissuance and non-reissuance restatements. In Panel B of Table 3, we partition each restatement measure based on breadth by using the median of the number of restatement reasons in the sample (two).^7^ The coefficient on *ERP_POST* is negative and significant for broad restatements (p<0.01) and non-reissuance restatements (p=0.09), but it is not significant for narrow restatements. For non-ERP-related restatements, coefficients of interest are positive and significant in four of six specifications. Specifically, the coefficient on *NON_ERP_POST* is positive and significant in all three columns that relate to narrow restatements and the coefficient is also positive and significant for broad reissuance restatements.

Overall, the results related to ERP-related acquisitions are consistent with insights of our interviewees. Specifically, the results are consistent with ERP-related expertise improving audit teams’ understanding of processes and testing of controls. The non-ERP-related acquisition results provide some evidence that shifts towards commercialism may result in an increase in narrower restatements (i.e., restatements that affect a few accounts or processes). However, our interviews did not suggest how non-ERP-related acquisitions should affect restatement breadth, so these results should be interpreted with caution.

### 3.4 Data analysis #4: are results due to PCAOB targeting offices that acquire consulting practices?

It is possible that the PCAOB increases engagement scrutiny from the offices that acquire consulting practices, and in particular, non-ERP consulting practices. If the PCAOB considers consulting acquisitions as a threat to the office culture and independence, then it is possible that related inspections would have produced a higher number of restatements and identified independence issues at these offices, which should be noted within inspection reports.^8^

To examine this possibility we gather PCAOB's inspection reports on each of the Big 4 firms issued on the years in our sample (2005-2015) and search for independence issues as follows. Using NVivo software we identified 315 instances containing the search term independen*.^9^ The majority of these instances (260 or approximately 83%) relate to the PCAOB's descriptions of its standards, scope, or inspection procedures. The second largest category relates to the PCAOB's descriptions of the procedures that the audit firm performed (24 or approximately 8%). The remaining categories included the firm's expression of commitment to independent audits (11 or approximately 3%), the PCAOB's description of the independent procedures (e.g., independent estimates) that the firm failed to perform entirely (eight, or approximately 2%) or sufficiently (six, or approximately 2%), and the PCAOB's description of the audit client's (the issuer's) procedures or operations (six, or approximately 2%). Given we find no instances of issues where the PCAOB believed that the firm's independence was compromised, it seems unlikely that our results are driven by PCAOB scrutiny of independence issues around acquisitions of consulting practices.^10^

To obtain additional context, we discussed the firms’ acquisitions of consulting practices and the extent to which PCAOB inspections incorporate information about non-audit services with three highly experienced PCAOB inspectors. The first two inspectors indicated that their consideration of any such activity is limited to the procedures of assessing the firm's independence of their audit clients. One of these two inspectors explained that “We would obviously consider if [non-audit services] would impair their independence… Part of what we would be concerned about is their independence process and making sure that they are independent of their audit clients. And that is our [only] concern.” The other of the two inspectors elaborated further: “[When] we do independence procedures… One of the things we get from firms is how much the audit fees are compared to tax fees compared to consulting fees… If the consulting fees are fairly high or [a] high percentage of the total fees that's obviously a risk indicator.” The third PCAOB inspector added that to their knowledge the firms do not need to gain approval from the PCAOB for acquisitions of consulting firms and do not need to report acquisitions of consulting firms to the PCAOB. Further, this inspector shared a belief that it would be reasonable for an audit firm to acquire a consulting practice to improve its expertise, particularly “if the firms are not [an] expert in those areas – they may want to start acquiring [expert consulting firms] or they will fall behind.”

We also asked a senior staff member at the PCAOB to comment on the absence of the independence issues in the inspection reports. The staff member commented as follows: “Consistent with Sarbanes-Oxley and the Board's rules, any criticisms of, or potential defects in, the quality control systems of a firm are non-public unless not addressed to the satisfaction of the Board within 12-months after the issuance of the Board's inspection reports. We have published information about independence matters identified during inspections in various speeches and inspection briefs.” Thus, it appears that the firms remediate any noted independence issues quickly and to the inspectors’ satisfaction, so that none of these issues were made public for any specific Big 4 firms during the period under our examination (2005-2015).

Overall, the results of our limited examination of PCAOB inspection reports and interviews of PCAOB inspectors do not suggest that during inspections the PCAOB targeted audit offices associated with acquisitions of consulting firms. Therefore, it seems unlikely that regulator scrutiny explains the positive association we find between non-ERP consulting firm acquisitions and audit quality, as measured by misstated financial statements.

**Table 4:** OLS regressions of audit quality measures by acquisition type of employees acquired at the firm level

Independent Variables	OLS Regressions, Dependent Variable is Listed in Row Below				
	*RESTATEMENTS*	*REISSUANCE_* *RESTATEMENTS*	*NON_REISSUANCE_* *RESTATEMENTS*	*ACCRUAL_* *QUALITY*	*ABNORMAL_* *ACCRUALS*
	(1)	(2)	(3)	(4)	(5)
*LOG_ERP_EMPLOYEES*	−0.007***	0.001	−0.008***	0.001*	0.000
	*0.002*	*0.001*	*0.002*	*0.000*	*0.000*
*LOG_NON_ERP_EMPLOYEES*	0.003***	0.001***	0.002*	0.000	0.000
	*0.001*	*0.001*	*0.001*	*0.000*	*0.000*
*LN_MVE*	−0.004	−0.002	−0.002	0.001	0.001
	*0.003*	*0.001*	*0.002*	*0.000*	*0.001*
*ROA*	0.019	0.015	0.003	−0.005	−0.037***
	*0.022*	*0.014*	*0.020*	*0.007*	*0.010*
*NEGATIVE_ROA*	0.013	0.004	0.008	−0.001	0.003
	*0.010*	*0.006*	*0.009*	*0.002*	*0.003*
*LEVERAGE*	0.040***	0.007	0.031***	−0.004	−0.006
	*0.012*	*0.007*	*0.011*	*0.003*	*0.004*
*BTM*	0.017***	0.004*	0.013***	−0.002***	−0.003***
	*0.003*	*0.002*	*0.003*	*0.001*	*0.001*
*FIN_RAISED*	0.014	0.017	0.003	0.030***	0.046***
	*0.019*	*0.012*	*0.017*	*0.004*	*0.005*
*STD_DEV_CFO*	−0.146***	−0.024	−0.140***	0.192***	0.231***
	*0.054*	*0.037*	*0.048*	*0.017*	*0.020*
*STD_DEV_SALE*	0.000	0.014	−0.011	0.026***	0.033***
	*0.019*	*0.012*	*0.018*	*0.004*	*0.005*
*LOSS_PROPORTION*	0.011	0.014*	0.007	0.013***	0.007*
	*0.015*	*0.009*	*0.014*	*0.003*	*0.004*
*INTANGIBLES*	0.003**	0.000	0.002	−0.002***	−0.003***
	*0.002*	*0.001*	*0.002*	*0.000*	*0.000*
*MERGER*	0.003	0.002	0.003	0.007***	0.008***
	*0.007*	*0.004*	*0.007*	*0.001*	*0.002*
*SALES_GROWTH*	0.004	0.005	−0.002	0.010***	0.021***
	*0.007*	*0.005*	*0.006*	*0.002*	*0.003*
*MW*	0.398***	0.340***	0.163***	0.007***	0.011***
	*0.016*	*0.018*	*0.018*	*0.002*	*0.003*
*404B_OPINION*	0.010	−0.006	0.013*	−0.004	−0.006*
	*0.011*	*0.008*	*0.010*	*0.003*	*0.003*
*PRIOR_RESTATEMENT*	0.051***	0.015***	0.036***	0.000	0.002
	*0.008*	*0.005*	*0.007*	*0.001*	*0.002*
*NON_BUSYSEASON*	0.002	0.004	0.000	−0.002	0.000
	*0.007*	*0.004*	*0.001*	*0.001*	*0.002*
Year Fixed Effects?	Yes	Yes	Yes	Yes	Yes
Industry Fixed Effects?	Yes	Yes	Yes	Yes	Yes
Auditor Office Fixed Effects?	No	No	No	No	No
Total Obs.	18,787	16,854	17,987	17,061	18,279
R^2^	0.09	0.16	0.05	0.15	0.14

This table reports the results of the following ordinary least squares regression:

*AUDIT_QUALITY* = β_0_+ β_1_*LOG_ERP_EMPLOYEES*+ β_2_*LOG_NON_ERP_EMPLOYEES* + β’*CONTROLS + Industry Fixed Effects + Year Fixed Effects +* ε

*AUDIT_QUALITY* represents one of the audit quality measures listed below in columns (1) thru (5). *LOG_ERP_EMPLOYEES (LOG_NON_ERP_EMPLOYEES)* is the log of the number of employees acquired related to (not related to) enterprise resource planning consulting or analytics consulting by firm in year *t.* Detailed variable definitions are provided in the Appendix. Standard errors are in italics and are clustered by company. *, **, and *** represent statistical significance at the 10%, 5%, and 1% levels, respectively, using a two-tailed test.

### 3.5 Data analysis #5: do audit quality effects from consulting firm acquisitions affect national-firm audit quality?

We focus our main analyses above and in the related research article at the audit office level rather than the national level because acquisition activity at a local office level is infrequent and more likely to be homogenous with respect to the focus of the acquisition (e.g. ERP vs. non-ERP). Additionally, the movement of consulting leadership and employees into a local office is more likely to be disruptive for an office rather than for the firm as a whole. Thus, we believe the audit quality effects are both more likely to occur and be detected at the office level. However, whether a national effect is present remains an interesting question that we explore.

To determine whether any national effects exist, we create national level ERP and non-ERP acquisition variables using the log of the sum of employees acquired from ERP (*LOG_ERP_EMPLOYEES)* and non-ERP *(LOG_NON_ERP_EMPLOYEES)* acquisitions in a given year by firm.^11^ Using company-year OLS regressions, we re-estimate equation (1) from the related research article replacing *ERP_POST* and *NON_ERP_POST* with the variables defined above and provide the results in Table 4.

Overall, we find evidence that is mostly consistent with our primary results. Specifically, the coefficient on the log of the number of ERP employees acquired in year *t* (*LOG_ERP_EMPLOYEES*) is negative and significant when the dependent variable is either *RESTATEMENTS* or *NON_REISSUANCE_RESTATEMENTS.* This suggests that the acquisition of ERP employees improves audit quality. However, the positive and weakly significant coefficient on *ACCRUAL_QUALITY* suggests the opposite. The coefficient on the log of the number of non-ERP employees acquired in year *t* (*LOG_NON_ERP_EMPLOYEES*) is positive and significant when the dependent variable is *RESTATEMENTS, REISSUANCE_RESTATEMENTS,* or *NON_REISSUANCE_RESTATEMENTS.* This suggests that the acquisition of non-ERP employees negatively affects audit quality. Thus, there is evidence that the audit quality effects found at the office level spill over to all offices within the firm consistent with the notion that consulting acquisitions may have an impact on the expertise and culture of the firm as a whole. We caution that the national office results are exploratory in nature, but could be explored further in future research.

## Declaration of Competing Interest

The authors declare that they have no known competing financial interests or personal relationships which have, or could be perceived to have, influenced the work reported in this article.

## Figures and Tables

**Table:** 

Variable	Definition and data source
Dependent variables	
*RESTATEMENT*	Coded 1 if year *t* is misstated for any reason, as subsequently revealed in a restatement announcement, and 0 otherwise (Audit Analytics).
*REISSUANCE_RESTATEMENT*	Coded 1 if year *t* is misstated via a reissuance as disclosed in an 8-k Item 4.02, as subsequently revealed in a restatement announcement, and 0 otherwise (Audit Analytics).
*NON_REISSUANCE_RESTATEMENT*	Coded 1 if year *t* is misstated for non-reissuance errors, as subsequently revealed in a restatement announcement, and 0 otherwise (Audit Analytics).
*ACCRUAL_QUALITY*	The absolute value of the residuals for year *t* from Dechow and Dichev (2002) as implemented in Barua et al. (2010) . The following regression is estimated cross-sectionally within each two-digit SIC code and year: (The following variables are from the Compustat database). TOTAL_ACCRUALS_it_ = β_0_ + β_1_*(OCF_it-1_) + β_2_*(OCF_it_) + β_3_*(OCF_it+1)_ + β_4_*(∆Sales_it_) + β_5_*(PPE_it_)+ ε where total accruals is calculated by: TOTAL_ACCRUALS*_it_* = ((ACT - lag ACT) - (CHE - lag CHE) - (LCT - lag LCT) + (DLC - lag DLC) - DP)/lag AT

**Table:** 

*ABNORMAL_ACCRUALS*	The absolute value of abnormal accruals for firm *i* in year *t* after subtracting off the median abnormal accrual from a performance-matched decile. The performance match is based off of return on assets in year *t.* Abnormal accruals is the error term for firm i in year t as measured using the following equation estimated by year and two-digit SIC code: (The following variables are from the Compustat database). TOTAL_ACCRUALS_it_ = β_0_ + β_1_*(1/ASSETS_it-1_) + β_2_*((∆REV_it_/ASSETS_it-1_) - (∆AR_it_/ASSETS_it-1_)) + β_3_*(PPE_it_/ASSETS_it-1_) + ε where total accruals is calculated by: TOTAL_ACCRUALS_it_ = (IB-OANCF)/lag AT.
*NUM_RESTATEMENT_REASONS*	The number of restatement codes identified by audit analytics for each restatement filed within the sample (Audit Analytics Non-Reliance Restatement database).
Independent variables	
*ERP_POST*	Coded 1 for the office-years audits with an audit opinion signature after the first consulting practice acquisition made by the audit firm where the acquired consulting practice's headquarters is in the same city as the audit office and where the consulting practice acquisition is related to either analytics consulting or enterprise resource planning consulting, and 0 otherwise. See Appendix A for a detailed listing of acquisitions where the consulting practice acquisition is related to either analytics consulting or enterprise resource planning consulting and 0 otherwise.
*NON_ERP_POST*	Coded 1 for the office-years audits with an audit opinion signature after the first consulting practice acquisition made by the audit firm where the acquired consulting practice's headquarters is in the same city as the audit office and where the consulting practice acquisition is not related to either analytics consulting or enterprise resource planning consulting, and 0 otherwise. See Appendix A for a detailed listing of acquisitions where the consulting practice acquisition is related to either analytics consulting or enterprise resource planning consulting and 0 otherwise.
*ERP_POST_tx*	*ERP_POST_tx* is an indicator set to 1 for any audit-office year audit with a signature *x* years following the first consulting practice acquisition made by the audit firm where the consulting firm's headquarters is in the city of the audit office and where the acquisition is related to enterprise resource planning or analytics consulting.

Variable	Definition and Data Source

*NON_ERP_POST_tx*	*NON_ERP_POST_tx* is an indicator set to 1 for any audit-office year audit with a signature *x* years following the first consulting practice acquisition made by the audit firm where the consulting firm's headquarters is in the city of the audit office and where the acquisition is related to enterprise resource planning or analytics consulting.
*LARGE_ERP_POST*	Coded 1 for the *ERP_POST_ACQUISITION_OFFICE* years where the number of employees acquired scaled by total audit fees for the related audit office in the year of the consulting acquisition is at or above the median of all consulting acquisitions, and 0 otherwise.
*SMALL_ERP_POST*	Coded 1 for the *ERP_POST_ACQUISITION_OFFICE* years where the number of employees acquired scaled by total audit fees for the related audit office in the year of the consulting acquisition is below the median of all consulting acquisitions, and 0 otherwise.
*LARGE_NON_ERP_POST*	Coded 1 for the *NON_ERP_POST_ACQUISITION_OFFICE* years where the number of employees acquired scaled by total audit fees for the related audit office in the year of the consulting acquisition is at or above the median of all consulting acquisitions, and 0 otherwise.
*SMALL_NON_ERP_POST*	Coded 1 for the *NON_ERP_POST_ACQUISITION_OFFICE* years where the number of employees acquired scaled by total audit fees for the related audit office in the year of the consulting acquisition is below the median of all consulting acquisitions, and 0 otherwise.
*LOG_ERP_EMPLOYEES*	The log of the number of employees acquired related to enterprise resource planning consulting or analytics consulting acquisitions by firm in year *t.*
*LOG_NON_ERP_EMPLOYEES*	The log of the number of employees acquired unrelated to enterprise resource planning consulting or analytics consulting acquisitions by firm in year *t.*
*NEGATIVE_ROA*	Coded 1 if (Compustat) ROA is less than 0, and 0 otherwise.

**Table:** 

*LEVERAGE*	A measure of financial leverage at the end of year *t.* Equal to the book value of interest-bearing debt scaled by total assets. (Compustat (DLTT+DLC)/AT).
*BTM*	Book to market measured by Compustat (CEQ/MKVALT).
*FIN_RAISED*	The sum of additional cash raised from the issuance of common and preferred stock and the issuance of long-term debt. This variable is deflated by average total assets. (Compustat (DLTIS+SSTK)/(Average AT))
*STD_DEV_CFO*	Standard deviation of cash flow from operations (Compustat OANCF) divided by total assets (Compustat AT), where the standard deviation is calculated using the prior five fiscal years, requiring a minimum of three years of data.
*STD_DEV_SALE*	Standard deviation of sales (Compustat SALE) divided by total assets (Compustat AT), where the standard deviation is calculated using the prior five fiscal years, requiring a minimum of three years of data.
*LOSS_PROPORTION*	The proportion in the last five years (*t-4* to *t)*of which the firm has reported a loss (Compustat IB<0).
*INTANGIBLES*	The natural log of one plus the total amount of intangible assets for year *t.* (Compustat INTAN).
*MERGER*	Coded 1 if a firm is involved in a merger or acquisition in year *t,* and 0 otherwise (Compustat SALE_FN).
*SALES_GROWTH*	Percentage growth of year over year sales revenue (Compustat (REVT_t_-REVT_t-1_)/REVT_t-1_).

Variable	Definition and Data Source

*MW*	Coded 1 if the auditor included a material weakness in internal control over financial reporting in the original internal control opinion for year *t,* zero otherwise (Audit Analytics SOX 404 Internal Controls database).
*404B_OPINION*	Coded 1 if the client receives an audit of internal control over financial reporting for year *t*, zero otherwise (Audit Analytics SOX 404 Internal Controls database).
*PRIOR_RESTATEMENT*	Coded 1 if a company discloses a prior-period restatement within the three years preceding the year *t* financial statements, zero otherwise (Audit Analytics Non-Reliance Restatements database).

## Footnotes

1 Searches were performed between 2/25/2015 and 3/1/2015.

2 The download was completed on 03/06/2018

3 There are 6 acquisitions of unknown size from our analyses. These include the acquisitions of Recombinant Capital, Bersin & Associates, Jeffrey A. Parker & Associates Inc., EntryPoint's Global Trade Management, HumanR, and Alaris Consulting. We consider these as small acquisitions for the sake of this study. Dropping these acquisitions from our analyses yields similar inferences.

4 A limitation of this analysis is that we cannot directly measure the number of consulting firm employees retained by the acquiring office.

5 We note that the coefficients and standard errors for *LARGE_NON_ERP_POST* and *SMALL_NON_ERP_POST* appear to be the same. However, the difference in statistical significance is due to rounding of the coefficient and standard error. The t-statistic and p-value for *LARGE_NON_ERP_POST* (*SMALL_NON_ERP_POST)* are 1.649 and 0.099 (1.390 and 0.166), respectively.

6 Caution should be exercised when considering the coefficients on *LARGE_ERP_POST* and *SMALL_ERP_POST* because the majority of the ERP acquisitions are relatively small. There are only four ERP acquisitions (Capital City Technologies, Market Point, Oco, Inc, and Optimum Solutions) that are considered large based on the median split of consulting firm employees divided by audit office total audit fees.

7 We include observations with two restatement codes in the narrower category; however, this creates a sample that is more heavily weighted with narrower restatements. Therefore, in untabulated sensitivity tests, we omit all observations with two restatement codes from the analysis and find similar results.

8 The PCAOB includes an assessment of independence within its inspections. For example, the PCAOB's report on Deloitte's 2011 inspection states the following (PCAOB [5], *emphasis* added): “This review addressed practices, policies and procedures concerning audit performance and the following five areas… (5) the Firm's processes for monitoring audit performance, including processes for identifying and assessing indicators of deficiencies in audit performance, *independence* policies and procedures, and processes for responding to weaknesses in quality control” (p. 21); and “The inspection team assessed policies, procedures, and guidance related to aspects of the firm's *independence* requirements and its consultation processes and the firm's compliance with them” (p. C-4).

9 Two coauthors independently coded each instance into one of the six categories. The Cohen's kappa statistic is 0.89, indicating a high level of agreement [4]. The coauthors resolved all 11 instances of disagreement.

10 One limitation of this analysis is that the PCAOB inspection report does not link deficiencies to audit offices. Therefore, we can only identify independence issues at the audit firm level and not the audit firm office level.

11 Note that data on number of employees is often a range (see Appendix A of the related research article). When this is the case, we use the top number of the range to create these two variables.
